# The Effects of Grandchild Care on Mental Health Among Chinese Elderly: The Mediating Effects of Social Networks

**DOI:** 10.1093/geroni/igaa057.1122

**Published:** 2020-12-16

**Authors:** Dan Tang, Jie Qiu, Kun Zhang

**Affiliations:** 1 Renmin University of China, Beijing, China; 2 Qingyuan Polytechnic, Qingyuan, China

## Abstract

Using the data of 2014 baseline survey of the China Longitudinal Aging Social Survey (CLASS), which provides a sample of older Chinese who had grandchild younger than 18 years old, this study examines the associations among grandchild care, social networks, and depressive symptoms among Chinese older adults. The older adults are divided into three groups basing on the frequency of their behaviors of taking care of grandchildren. The three groups are ‘no care, providing care occasionally, providing care frequently’. The mediating and moderating effects of social networks between grandchild care and depressive symptoms are tested. Results show that older adults who provide grandchild care report superior social networks and better mental health than those who don’t provide grandchild (reference group). After controlling the related variables, the older adults who provide grandchild occasionally benefit more than those who take care of grandchild frequently. Grandchild care is related to larger social networks, and the social networks are fully mediating the association between grandchild care and depressive symptoms.

